# Antioxidant Responses and Growth Impairment in *Cucurbita moschata* Infected by *Meloidogyne incognita*

**DOI:** 10.3390/biology13040267

**Published:** 2024-04-16

**Authors:** Yuh Tzean, Kuang-Teng Wang, Elena Gamboa Chen, Hung-Wen Wang, Tsung-Meng Wu, Chia-An Liu

**Affiliations:** 1 Department of Plant Medicine, National Pingtung University of Science and Technology, Pingtung 91201, Taiwan; lunacat621@gmail.com (E.G.C.); b0981099276@gmail.com (H.-W.W.); anis351321@gmail.com (C.-A.L.); 2 Department of Aquaculture, National Pingtung University of Science and Technology, Pingtung 91201, Taiwan; p10913002@mail.npust.edu.tw (K.-T.W.); wzm@mail.npust.edu.tw (T.-M.W.); 3 Institute of Biotechnology, National Taiwan University, Taipei 10617, Taiwan

**Keywords:** pumpkin, *Cucurbita moschata*, root-knot nematodes, *Meloidogyne incognita*, oxidative stress, antioxidative enzymes, plant–nematode interaction

## Abstract

**Simple Summary:**

Pumpkins (*Cucurbita moschata*), valued for their nutritional, medicinal, and economic contributions, are threatened by root-knot nematodes, notably *Meloidogyne incognita*. This research explores the impact of *M. incognita* on the growth and comprehensive physiological responses of pumpkins. The findings reveal that infection leads to significant growth impairment, as indicated by reduced plant height and biomass along with the development of nematode-induced galls. In addition, there is an observable oxidative stress response characterized by elevated levels of hydrogen peroxide and an increase in antioxidant defense mechanisms such as crucial antioxidative enzymes (superoxide dismutase, glutathione reductase, and catalase) and the accumulation of glutathione. These responses demonstrate a dynamic interplay between the plant and the nematode, where pumpkins mobilize robust antioxidant defenses to counteract the stress induced by nematode infection. Despite these defense mechanisms, pumpkin’s ability to combat *M. incognita* raises concerns about the agricultural production challenges posed by this pest in Cucurbita crops. The insights gained from this study improve our understanding of plant–nematode interactions, paving the way for strategies aimed at increasing resistance against these pests, thus promoting sustainable agricultural practices.

**Abstract:**

Pumpkins (*Cucurbita moschata*), valued for their nutritional, medicinal, and economic significance, face threats from *Meloidogyne incognita*, a critical plant-parasitic nematode. This study extensively examines the impact of *M. incognita* on the growth, physiological, and biochemical responses of *C. moschata*. We demonstrate that *M. incognita* infection leads to significant growth impairment in *C. moschata*, evidenced by reduced plant height and biomass, along with the significant development of nematode-induced galls. Concurrently, a pronounced oxidative stress response was observed, characterized by elevated levels of hydrogen peroxide and a significant increase in antioxidant defense mechanisms, including the upregulation of key antioxidative enzymes (superoxide dismutase, glutathione reductase, catalase, and peroxidase) and the accumulation of glutathione. These responses highlight a dynamic interaction between the plant and the nematode, wherein *C. moschata* activates a robust antioxidant defense to mitigate the oxidative stress induced by nematode infection. Despite these defenses, the persistence of growth impairment underscores the challenge posed by *M. incognita* to the agricultural production of *C. moschata*. Our findings contribute to the understanding of plant–nematode interactions, paving the way for the development of strategies aimed at enhancing resistance in Cucurbitaceae crops against nematode pests, thus supporting sustainable agricultural practices.

## 1. Introduction

The Cucurbitaceae family is a significant source of nutritionally and medicinally valuable plants. In addition to their nutritional and medicinal importance, cucurbits are esteemed for their aesthetic, cultural, medicinal, and botanical significance [1]. It encompasses 2 subfamilies, 118 genera, and over 800 species [2,3] and has been intertwined with human culture and dietary practices for over 12,000 years, making it an essential part of diverse and nutritious diets worldwide [1,4,5]. Despite originating in Asia, the Cucurbitaceae family has had numerous long-distance dispersal events, leading to its global distribution and economic importance across various continents [6]. The characteristic members of Cucurbitaceae include fruits, such as melon (*Cucumis melo*) and watermelon (*Citrullus lanatus*), and major vegetables, such as cucumber (*C. sativus*), zucchini (*Cucurbita pepo*), and pumpkin (*C. maxima*, *C. moschata*, and *C. argyrosperma*) [7,8]. Today, cucurbits rank among the major fruits and vegetables grown worldwide in both indoor and open-field settings [4,9].

Among these cucurbits, pumpkin is well-regarded as a versatile crop with significant implications for food security and sustainable agricultural practices. Notably, pumpkins are esteemed for their ability to produce some of the largest fruits among flowering plants and their rich composition of essential nutrients, including a diverse array of amino acids critical for human health [1,10,11]. These attributes make them suitable for a wide range of applications in food and feed. In addition to their application in food and feed, pumpkin has been historically utilized in folk medicine for managing gastrointestinal diseases [12], and its seeds contain unsaturated fatty acids, phenolic compounds, tocopherols, and minerals, which enhance their potential as functional ingredients [13,14]. Together, pumpkins and their seeds offer a plethora of nutritional and medicinal benefits that can be harnessed in various applications, from functional foods to nutraceuticals.

Pumpkins, despite their utility and nutritional value, are susceptible to various pathogens and pests, including plant–parasitic nematodes (PPNs). Plant–parasitic nematodes pose a significant threat to global food security. There are approximately 4300 known species of PPNs, accounting for 7% of the phylum Nematoda [15]. It has been reported that PPN infection causes an annual global loss of over 157 billion dollars, making it one of the most invasive types of diseases affecting plants [16].

Among PPNs, the root-knot nematode (RKN, *Meloidogyne* spp.) is one of the most important and damaging pests in agriculture [17]. Root-knot nematodes are sedentary endoparasitic nematodes that induce pathological changes in plant root systems [18]. These sedentary endoparasitic nematodes establish a complex and intimate relationship with their host plants, leading to the redifferentiation of vascular cells into large multinucleate feeding cells for an extended period, often lasting more than one month [19]. The process of giant cell formation involves the enlargement of cells and their conversion into multinucleate structures through synchronous nuclear divisions without cell division. This phenomenon, known as hypertrophy, is accompanied by hyperplasia of the surrounding root cells, which contributes to the formation of the characteristic root galls [20]. The hypertrophied giant cells and the hyperplastic root cells disrupt the normal architecture and function of the plant’s vascular system. This intricate interaction between root-knot nematodes and plant roots not only undermines the structural integrity and functionality of the plant’s vascular system but also significantly hampers its overall health and productivity, posing a substantial challenge to agricultural sustainability.

The widespread prevalence and diverse species of RKNs present a significant challenge to important horticultural crops such as pumpkins, with particular species inflicting distinct patterns of damage and stress. Comprising 98 species, RKNs affect most vascular plants and cause significant agricultural concern [17]. The most notable species, referred to as the four major species, include *M. arenaria*, *M. hapla*, *M. javanica*, and *M. incognita* [21]. These infestations typically result in a range of detrimental effects: yield reductions are commonly marked by observable symptoms such as root galls, stunted plant development, and premature wilting, which directly impact agricultural productivity [22,23,24]. Moreover, RKN infection can compromise the plant’s immune system, making it more susceptible to secondary infections from other pathogens, further exacerbating the detrimental impact on crop health and reducing yield [25]. This confluence of direct and indirect consequences of RKN activity underscores the urgency for targeted research and innovative management approaches to mitigate their pervasive impact.

An important aspect of controlling and managing RKNs in pumpkin cultivation is understanding the interaction between RKNs and plant responses. Studies have shown that plants activate a defense mechanism when affected by nematode pathogenesis, using reactive oxygen species (ROS) as antimicrobial agents and signaling molecules [26,27,28]. However, excessive ROS can cause irreversible damage to proteins, lipids, and nucleic acids, leading to cellular mortality [29]. To counteract these effects, plants have developed an antioxidant system composed of enzymes and various antioxidants, including superoxide dismutase (SOD), catalase (CAT), peroxidase (POD), and ascorbate peroxidase (APX) [30]. In addition, intrinsic antioxidants, such as ascorbic acid (ASC) and reduced glutathione (GSH), play a pivotal role in neutralizing the adverse effects caused by ROS [31]. These antioxidative defenses are critical for maintaining cellular homeostasis against damage from RKN infection.

The understanding and assessment of the modulation of ROS activities and antioxidative enzymes in pumpkin–RKN interactions provide insights into potential strategies for managing and mitigating the detrimental effects of the infection. By examining the antioxidant response and growth impairment in *C. moschata* infected by *M. incognita*, we aim to gain a better understanding of how the antioxidant system in plants functions in response to oxidative stress caused by RKN infection. We investigated ROS levels, particularly focusing on the roles of ROS and the antioxidant enzymes that regulate them. The present study provides comprehensive insights into the impact of *M. incognita* on *C. moschata*, including analyses of growth, ROS substances, and antioxidative enzyme activity.

## 2. Materials and Methods

### 2.1. Experimental Materials

In this study, pumpkin, *Cucurbita moschata* var. Er-Gu seedlings (Known-You Seed Co., Ltd., Kaohsiung, Taiwan), was selected as the test plant for the experiments. The test plants were grown in a growth medium consisting of a blend of peat and vermiculite (4:1 ratio) and maintained in a covered greenhouse. After reaching the two-leaf stage, the plants were transplanted in 3-inch pots for mock- or *Meloidogyne incognita* (Mi)-inoculation. For the Mi-inoculated group, plants were transplanted to 300 g of soil containing 3000 *M. incognita* juveniles. For the mock control group, plants were transplanted in soil without *M. incognita* exposure to serve as a baseline for comparison. The plants were collected at 42 days post-inoculation (dpi) for subsequent detailed growth measurements and analysis of reactive oxygen species (ROS) substances, and antioxidative enzyme activity.

### 2.2. Growth Parameter Measurements

At 42 dpi, various growth parameter measurements were taken to assess the impact of *M. incognita* on *C. moschata* plants. These evaluations include imaging the plant phenotypes, measuring mock- and *M. incognita*-inoculated plant height, root length, shoot weight, and root weight, and assessing the average gall numbers and galling index according to Zeck’s scale [32]. For plant height, the plant shoots were measured from the soil as the baseline. For root length measurement, the roots of the plants were washed with running water and imaged for subsequent processing by ImageJ software 1.53t [33]. The use of scale bars and color palette cards ensured consistency among images. Additionally, to assess the impact of *M. incognita* on *C. moschata*, the shoot and root sections of each plant were separated and weighed. The number of root-knot nematode galls was counted, and the galling index of mock- or *M. incognita*-inoculated plants was determined by Zeck’s scale [32] as an indicator of infection severity. According to Zeck’s scale, a rating of 0 indicates an uninfected plant root system, while a rating of 10 denotes a severely damaged plant with a completely compromised root system [32].

### 2.3. Evaluation of Hydrogen Peroxide (H_2_O_2_), Superoxide Radical (O_2_^•−^), and Malondialdehyde (MDA) Levels

The assessment of H_2_O_2_ levels was performed following a modified approach based on a previously described method [34]. This involved utilizing the extinction coefficient of 0.28 μmol^−1^cm^−1^ to calculate its concentration. The quantification of O_2_^•−^ was adapted from the methodology previously described [35], involving the conversion of hydroxylamine to nitrate as an indicator of O_2_^•−^ presence. A standard curve was created from varying concentrations of sodium nitrite ranging from 0 to 10 µM in order to measure O_2_^•−^. Lipid peroxidation was estimated by measuring malondialdehyde (MDA) levels using a procedure that has been previously described [36]. This process included extracting MDA using a 5% (*w*/*v*) trichloroacetic acid solution and then quantifying it through the thiobarbituric acid reaction to determine lipid peroxidation levels accurately for further analysis and comparison with known standards.

### 2.4. Analysis of Ascorbate (ASC) and Glutathione (GSH) Content

The quantification of ascorbate (ASC) levels involved a spectrophotometric approach, utilizing the chromatic transition that occurs when ASC reduces iron from Fe^3+^ to Fe^2+^. This Fe^2+^ form subsequently reacts with α,α′-dipyridyl to produce a colored complex detected at 525 nm. Total ascorbate content was determined by first converting dehydroascorbate (DHA) to ASC using dithiothreitol (DTT), with quantification against a standard curve of ascorbate [37]. Subsequently, the absorbance values were used for calculation according to Beer-Lambert law and compared with the reference standards.

For the GSH assay [38], approximately 0.1 g of plant material was processed in a 5% (*v*/*v*) trichloroacetic acid (TCA) solution. The mixture was then centrifuged 12,000× *g* for 10 min at 4 °C to obtain a clear extract. This acidic extract was neutralized with sodium phosphate buffer (0.4 M, pH 8.0) and divided into aliquots for total GSH and oxidized glutathione (GSSG) measurement using an enzymatic recycling technique involving the conversion of GSH in the presence of 5,5′-dithiobis (2-nitrobenzoic acid) and subsequent reduction by NADPH catalyzed by glutathione reductase (GR). The absorbance readings were taken at 412 nm wavelength. For specific GSSG determination, 2-vinylpyridine was incorporated into the samples. Calibration was accomplished using standard solutions of GSH and GSSG.

### 2.5. Antioxidative Enzyme Activity Assays

Antioxidative enzyme activities were assessed by homogenizing plant root tissues in a 0.1 M sodium phosphate buffer (pH 7.0) using a chilled pestle and mortar. The protein concentrations in enzyme extracts were determined using an adapted Bradford assay [39], which is a colorimetric technique for protein quantification and concentration determination. Briefly, a calibration curve was established using serial dilutions of high-grade BSA to fit within the assay’s operational concentration range (125–1000 μg/mL). For the assay, Coomassie brilliant blue reagent (Bio-Rad Laboratories Inc., Hercules, CA, USA) (1 mL) was added to blank, BSA standard, or protein samples (20 μL in a test tube). After thorough mixing with a vortex and a 30-min incubation at ambient temperature, the absorbance of the mixture at 595 nm was recorded in triplicate using a spectrophotometer (model: U-5100, Hitachi, Tokyo, Japan).

For assays involving ascorbate peroxidase (APX), the extraction medium was supplemented with 2 mM of ascorbate. The protocol for APX activity followed a modified version of the previously outlined procedure [40], with each unit of activity corresponding to the consumption of 1 nmol of ascorbate per minute under the assay conditions.

The evaluation process for glutathione reductase (GR) activity involved utilizing a revised adaptation of the methodology reported previously [41]. One unit of activity was defined as the quantity of enzyme that catalyzes the transformation of 1 µmol of β-NADPH per minute.

To measure superoxide dismutase (SOD) activity, we adopted a previously described method [42], where one unit of activity was equated to the enzyme amount causing 50% inhibition of the nitro blue tetrazolium (NBT) reduction, monitored at 560 nm, compared to a nonenzyme-containing blank.

For catalase (CAT) activity quantification, the assay was conducted based on a previously established method [43]. One unit of activity was defined as the enzyme quantity that decomposes 1 μmol of H_2_O_2_ per minute.

The peroxidase (POD) activity was assessed by conducting a spectrophotometric analysis. This involved the reaction of H_2_O_2_ with guaiacol to produce the oxidized product, tetraguaiacol, resulting in a measurable change in absorbance at the 470 nm wavelength [43]. One unit of POD activity was defined as the amount of enzyme required to facilitate the production of 1 µmol of tetraguaiacol per minute, per milligram of protein.

### 2.6. Sodium Dodecyl Sulfate-Polyacrylamide Gel Electrophoresis (SDS-PAGE) and Native Gel Enzymatic Activity Assay

Root samples (0.1 g) were meticulously homogenized in a 50 mM sodium phosphate buffer (pH 7.0), augmented with 2 mM Na_2_EDTA and 1 mM phenylmethylsulfonyl fluoride (PMSF) to inhibit protease activity. The resultant homogenate was then centrifuged at 13,000× *g* for 15 min at a controlled temperature of 4 °C to separate the supernatant, which contained the enzymatic proteins.

The protein separation via SDS-PAGE followed the protocol previously established [44], utilizing a 12% resolving-polyacrylamide gel. Proteins for electrophoresis were prepared by mixing the extracted proteins (8 μg for constant protein and 45 μL for constant volume) with bromophenol blue and glycerol, excluding sodium dodecyl sulfate (SDS) for native PAGE applications. A 10% resolving gel was employed for subsequent analyses.

For APX activity analysis, the extraction buffer was supplemented with 2 mM ascorbate. APX zymography was executed at 4 °C using a running buffer also containing 2 mM ascorbate. Post-electrophoresis, APX activity was visualized via staining as previously described [45].

Peroxidase activity was assessed following the procedure described previously [46]. Briefly, the gel was rinsed with distilled water to eliminate residual running buffer, then incubated in a staining mixture comprising 4.5 mM guaiacol and 22.5 mM H_2_O_2_ in 100 mM phosphate buffer (pH 7.0) at room temperature (25 °C).

GR activity was quantified using the staining protocol described previously [47]. The gel was stained in a solution containing 250 mM Tris-HCl (pH 7.5), 3 mM Na_2_EDTA, 0.4 mM NADPH, 0.68 mM 2,6-dichlorophenolindophenol (DCIP), 0.48 mM 3-(4,5-dimethyl-2-thiazolyl)-2,5-diphenyl-2H tetrazolium bromide (MTT), and 3.4 mM GSSG. A duplicate gel was processed without GSSG as a negative control.

The methodology for determining SOD activity was adopted from a previously reported protocol [42]. The gel was first immersed in a 2.45 mM nitroblue tetrazolium (NBT) solution for 15 min, followed by incubation in a 50 mM sodium phosphate buffer (pH 7.8) containing 28 mM riboflavin and 28 mM tetramethylethylenediamine (TEMED) in dark conditions for 15 min. Subsequent exposure to light for 15 min allowed for the visualization of SOD activity. To discern SOD isoenzymes, gels were pretreated with either 8 mM KCN or H_2_O_2_ in 50 mM sodium phosphate buffer (pH 7.0) prior to SOD staining. The staining of proteins within the gel was conducted using Coomassie blue staining to enable the visualization of protein bands.

## 3. Results

### 3.1. Effect of Meloidogyne incognita on Relative Growth Rate and Physiological Features of Cucurbita moschata

**Figure 1:**
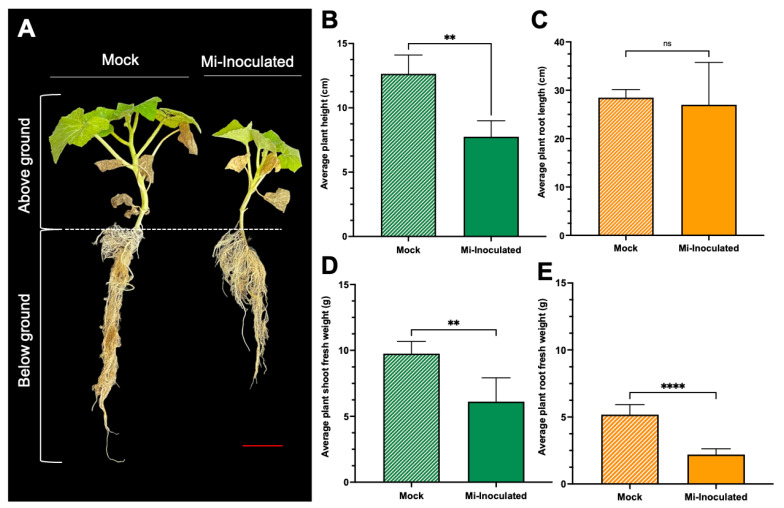
The impact of root-knot nematodes (RKN, *Meloidogyne incognita*) on the relative growth and physiological characteristics of pumpkin (*Cucurbita moschata*). (**A**) A photograph showing the phenotype of the *C. moschata* plant taken 42 days post-inoculation (dpi). *Meloidogyne incognita*-inoculated plants are labeled as Mi-inoculated; the scale bar represents 5 cm. Quantification of the average plant height (**B**) and the average root length (**C**). Quantification of the average plant shoot weight (**D**) and average plant root weight (**E**). The values represent mean  ±  SE (*n*  =  5); ** indicates *p*  ≤  0.01; **** indicates *p*  ≤  0.0001; and ns denotes no statistical significance. The Student’s *t* test compares groups as indicated.

**Figure 2:**
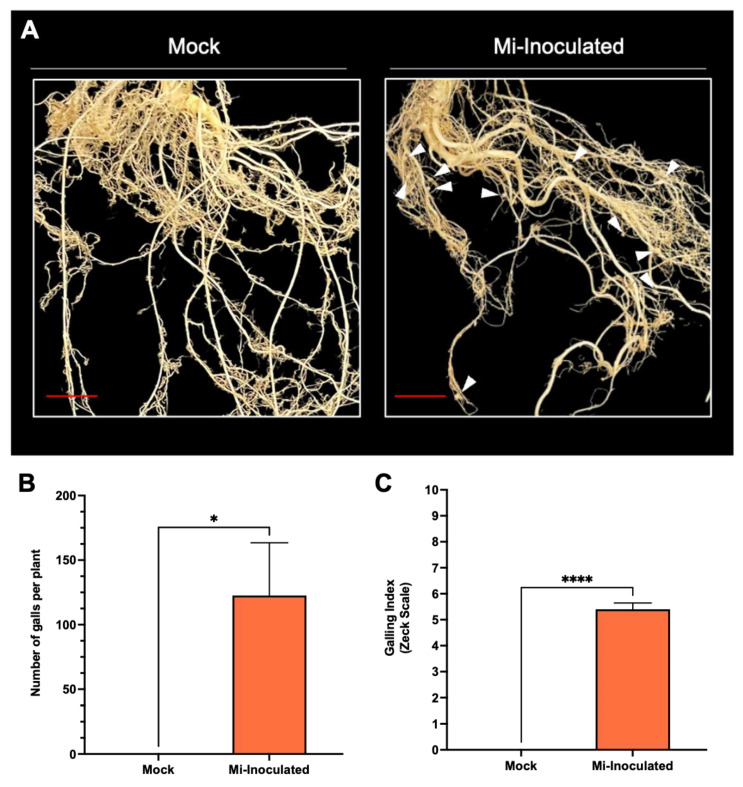
The comparative evaluation of the pumpkin (*Cucurbita moschata*) root system in response to root-knot nematode (RKN, *Meloidogyne incognita*) inoculation. (**A**) Presence of RKN galls on roots seen in *C. moschata* at 42 days post-inoculation (dpi). *Meloidogyne incognita*-inoculated plants are labeled as Mi-inoculated; the scale bar represents 1 cm; white arrows indicate observed galls. (**B**) Measurement for quantifying the number of RKN galls per plant on the roots of *C. moschata* at 42 dpi. The values represent mean  ±  SE (*n*  =  5); * indicates *p*  ≤  0.05. The Student’s *t* test compares groups as indicated. (**C**) The severity of gall formation was assessed according to Zeck’s [32] 0–10 scale, where 0 represents no galls, while a score of 10 indicates root necrosis attributed to *M. incognita* infection. The values represent mean  ±  SE (*n*  =  5); **** indicates *p*  ≤  0.0001. The Student’s *t* test compares groups as indicated.

*Cucurbita moschata* plants were infected with *M*. *incognita* to assess the impact on plant growth and development phenotypes. At 42 days post-inoculation (dpi), it was observed that *M. incognita*-infected pumpkins exhibited significantly lower plant height compared to mock control, as depicted in Figure 1A,B. Additionally, although no significant difference was noted in root length between mock control and *M. incognita*-infected plants (Figure 1C), the average plant fresh weight of both shoot and root for the infected plants was found to be significantly lower compared to mock control plants (Figure 1D,E). Analysis of roots from *M*. *incognita*-infected plants (Figure 2) revealed an average of 122 root-knot nematode (RKN) galls per plant (Figure 2B) with a galling index score of 5.4 according to the Zeck’s scale (Figure 2C) [32], suggesting the pronounced development of RKN symptoms at 42 dpi in *C. moschata*. The results indicate that infection with *M. incognita* has a detrimental effect on the growth and development of *C. moschata*.

### 3.2. Investigating the Impact of Meloidogyne incognita on Hydrogen Peroxide (H_2_O_2_), Superoxide Radical (O_2_^•−^), and Malondialdehyde (MDA) Concentrations

**Figure 3:**
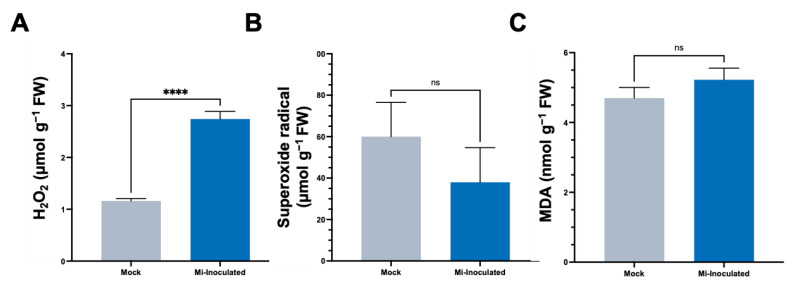
The analysis of stress-related parameters, including hydrogen peroxide (H_2_O_2_) (**A**), superoxide radical (O_2_^•−^) (**B**), and malondialdehyde (MDA) (**C**) content in the roots of pumpkin (*Cucurbita moschata*) under mock or *Meloidogyne incognita*-inoculated (Mi-inoculated) conditions at 42 days post-inoculation. The values represent mean  ±  SE (*n*  =  5); **** indicates *p*  ≤  0.0001; and ns denotes no statistical significance. The Student’s *t* test compares groups as indicated.

In the subsequent analyses, we evaluated the impact of *M. incognita* on various oxidative stress parameters, including hydrogen peroxide (H_2_O_2_) concentrations, the production of superoxide radicals (O_2_^•−^), and quantities of malondialdehyde (MDA) (Figure 3). The results showed that the *M. incognita*-inoculated group had a significant 2.4-fold increase in H_2_;O_2_ levels compared to the mock control group (Figure 3A). Conversely, we found the average O_2_^•−^ concentration was lower in the *M. incognita*-inoculated plants compared to the mock control group; however, based on our analysis results (Figure 3B), this difference did not reach statistical significance. Evaluation of MDA levels as an indicator of lipid peroxidation and correlation with reactive oxygen species (ROS) levels revealed no significant difference between the mock control and *M. incognita*-inoculated group (Figure 3C).

### 3.3. Effect of Meloidogyne incognita on Ascorbate (ASC) and Glutathione (GSH) Levels in Cucurbita moschata

**Figure 4:**
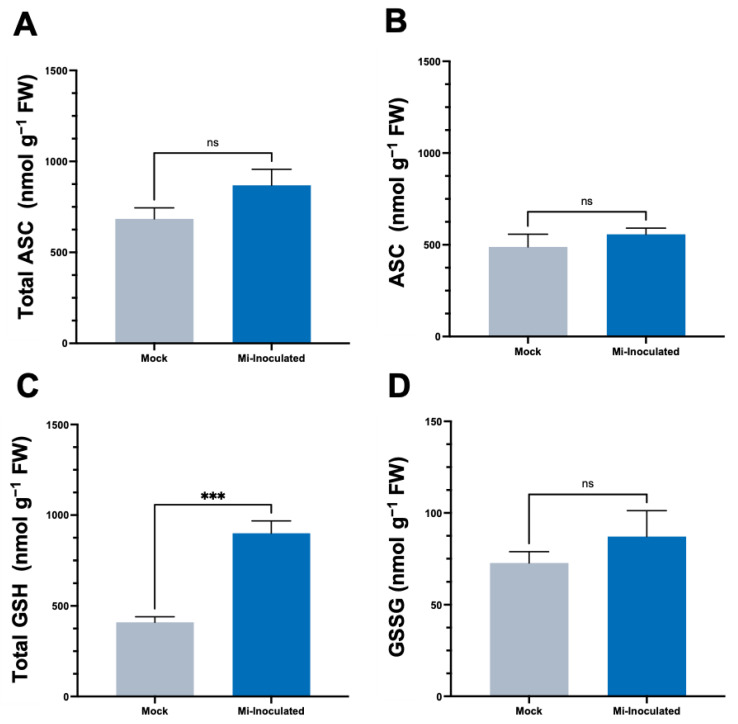
Effects of *Meloidogyne incognita* on the levels of total ascorbate (**A**), ASC (**B**), total glutathione (**C**), and GSSG (**D**) in *Cucurbita moschata*. *Meloidogyne incognita*-inoculated plants are labeled as Mi-inoculated. The values represent mean  ±  SE (*n*  =  5); *** indicates *p*  ≤  0.001; and ns denotes no statistical significance. The Student’s *t* test compares groups as indicated.

We conducted further analysis to examine the impact of RKN *M. incognita* on antioxidant molecules within *C. moschata*, specifically targeting ascorbate (ASC) and glutathione (GSH) concentrations. Our observations at 42 dpi showed that although there was an increase in the levels of both total ASC and total GSH (Figure 4), a statistically significant elevation (2.2-fold) of total GSH levels was only found in the *M. incognita*-inoculated group compared to the mock control group (Figure 4C).

### 3.4. Assessment of Protein Levels and Antioxidative Enzyme Activity in Cucurbita moschata Post-Inoculation with Meloidogyne incognita

**Figure 5:**
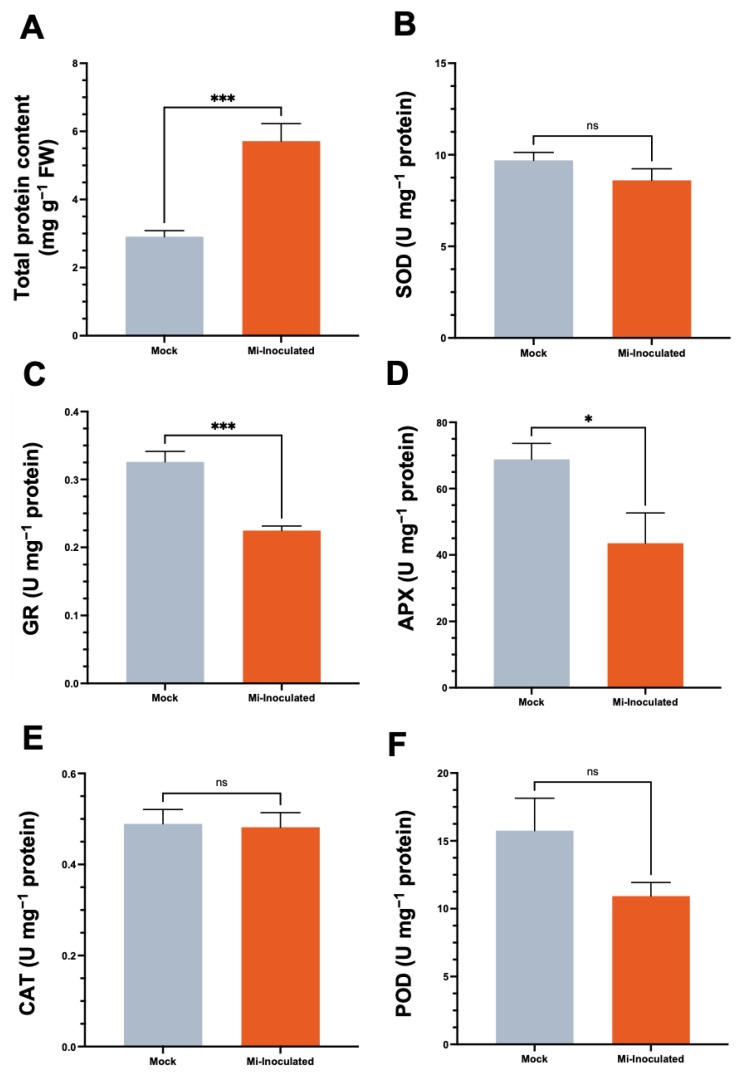
Impact of *Meloidogyne incognita* on total protein content (**A**) and antioxidative enzyme activities, including superoxide dismutase (SOD) (**B**), glutathione reductase (GR) (**C**), ascorbate peroxidase (APX) (**D**), catalase (CAT) (**E**), and peroxidase (POD) (**F**) in *Cucurbita moschata*, normalized to protein content. *Meloidogyne incognita*-inoculated plants are labeled as Mi-inoculated. The values represent mean  ±  SE (*n*  =  5); * indicates *p*  ≤  0.05; *** indicates *p*  ≤  0.001; and ns denotes no statistical significance. The Student’s *t* test compares groups as indicated.

We examined the total protein concentration and specific activities of antioxidative enzymes, including superoxide dismutase (SOD), glutathione reductase (GR), catalase (CAT), and peroxidase (POD), in *C. moschata* exposed to *M. incognita* infection at 42 dpi (Figure 5). Our analysis showed that although a 2.0-fold increase in protein content was observed in the *M. incognita*-infected group compared to the mock control group (Figure 5A), the GR activity in *C. moschata* was significantly lower (0.69-fold) compared to the mock control (Figure 5C). In addition, following the inoculation with *M. incognita*, APX exhibited a decrease of 0.63-fold compared to the mock control (Figure 5D). No significant difference was observed for SOD (Figure 5B), CAT (Figure 5E), and POD (Figure 5F). Conversely, when normalizing the enzyme activities to fresh weight, the overall SOD activity in the *M. incognita*-inoculated group was significantly enhanced, with a 1.7-fold increase compared to the mock control (Figure S1A). Similarly, glutathione reductase (GR) activity in *C. moschata* was significantly higher following inoculation with *M. incognita*, exhibiting a 1.6-fold increase compared to the control (Figure S1B). CAT and POD activities also increased in response to *M. incognita* by 1.9-fold and 1.6-fold, respectively, relative to the mock control (Figure S1D,E).

### 3.5. Assessment of Antioxidative Enzyme Isoforms in Cucurbita moschata via Native Gel Activity Assay following Meloidogyne incognita Inoculation

**Figure 6:**
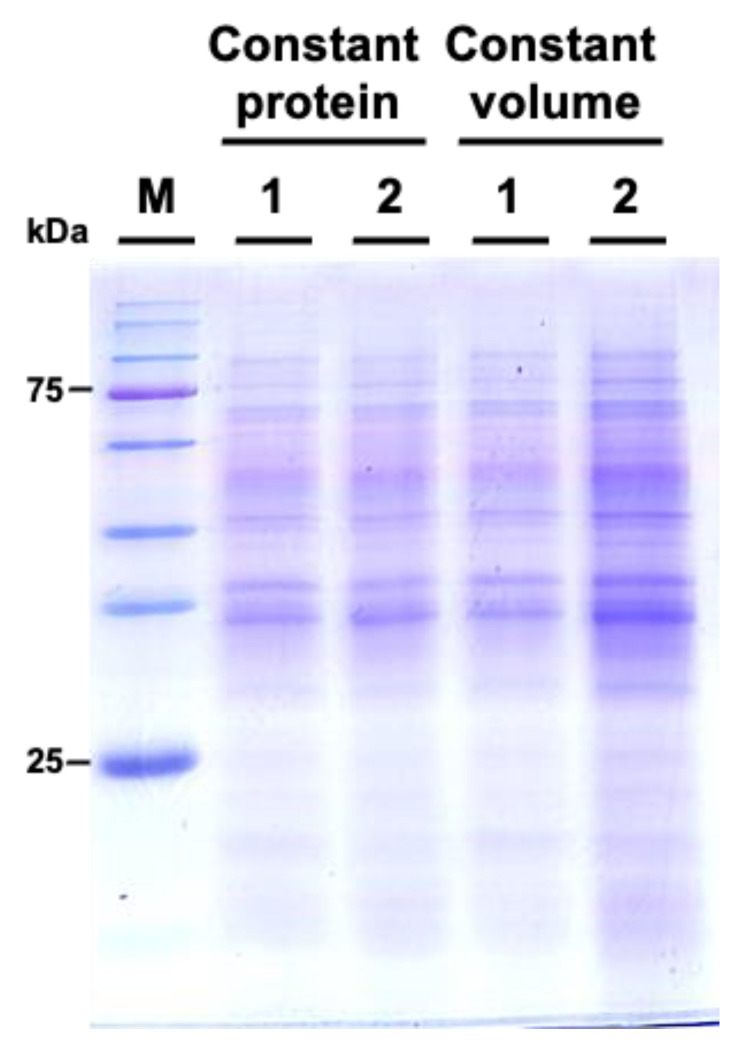
Comparative SDS-PAGE analysis of protein profiles from *Cucurbita moschata* subjected to mock treatment and *Meloidogyne incognita* infection. 1 represents proteins from mock-treated plants, and 2 represents proteins from *M. incognita*-infected plants. Constant protein or constant volume indicates whether the loading of protein samples is normalized to total protein (8 μg) or volume (45 μL).

We subsequently examined the protein profiles by using sodium dodecyl-sulfate polyacrylamide gel electrophoresis (SDS-PAGE). The results indicated that the protein patterns between *M. incognita*-inoculated and mock control groups showed similar patterns (Figure 6).

**Figure 7:**
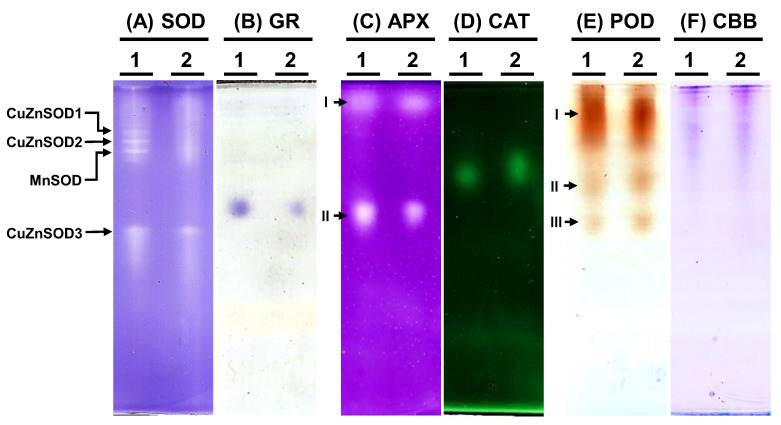
Native polyacrylamide gel (PAGE) analysis of antioxidative enzyme activities in *Cucurbita moschata* infected by *Meloidogyne incognita*. A total of 8 μg of proteins was loaded in each lane. (A) Superoxide dismutase (SOD) isozymes, (B) glutathione reductase (GR) isozymes, (C) ascorbate peroxidase (APX) isozyme, (D) catalase (CAT) isozyme, (E) peroxidase (POD) isozymes, and (F) Coomassie Brilliant blue staining (CBB) for protein quantification. 1, mock; 2, *Meloidogyne incognita* (Mi)-inoculated. I, II, and III indicate different isoforms.

To examine the antioxidative enzyme isoforms within *C. moschata*, native polyacrylamide gel electrophoresis (PAGE) was employed to discern the presence of SOD, GR, APX, CAT, and POD variants (Figure 7). Analysis revealed the existence of four distinct SOD isoforms in *C. moschata* (Figure 7A). Treatment with specific inhibitors enabled the differentiation of these isoforms, identifying them as three copper-zinc SODs (CuZnSODs) and one manganese SOD (MnSOD). Iron SOD (FeSOD) was absent in *C. moschata*. For GR, a single band was discernible (Figure 7B), while in terms of APX isoforms, two variants (APX-I and APX-II) were detected (Figure 7C). Furthermore, a single band was observed for CAT activity (Figure 7D). POD isoform analysis, conducted with guaiacol and H_2_O_2_, revealed three POD variants in *C. moschata* (Figure 7E). Equal protein loading across all lanes was verified (Figure 7F).

## 4. Discussion

Pumpkins (*C. moschata*) and their derivatives are important for their nutritional, medicinal, and economic values. However, PPN continues to pose a significant constraint on agricultural systems. RKNs, belonging to the genus *Meloidogyne*, are one of the most important groups of PPNs worldwide. The present study delineates the physiological and biochemical responses of *C. moschata* to *M. incognita* infection. Our findings reveal that *M. incognita* triggers significant alterations in growth parameters, oxidative stress indicators, and antioxidative defense responses, illuminating the intricate interplay between *C. moschata* physiology and *M. incognita*-induced stress adaptations.

Cucurbit crops are susceptible to root-knot nematodes. A previous evaluation of six cucurbit crops (squash, cucumber, cantaloupe, watermelon, smooth luffa, and angled luffa) revealed that among the different *Meloidogyne* spp. (*M. enterolobii*, *M. floridensis*, *M. hapla*, *M. incognita*, and *M. javanica*), *M. incognita* was found to be more damaging [48]. This pathogen is documented to exert detrimental effects across cucurbits, with pumpkins being notably vulnerable compared to cucumber, melon, squash, and watermelon [49]. Our investigation into the infection of *C. moschata* by *M. incognita* revealed significant impairments in growth, as evidenced by marked reductions in plant height and fresh weight, along with notable galling indices (Figure 1 and Figure 2). These findings are consistent with previous reports, further substantiating the detrimental effects of nematode infections on plant vigor and productivity [50,51,52,53]. At higher nematode inoculation levels compared to this study, the galling indices of different susceptible *C. moschata* genotypes could reach 8.0 to 8.25 [54]. Therefore, implementing timely management practices to counter nematode exposure is crucial in mitigating their detrimental impacts, emphasizing the importance of strategic interventions in preserving cucurbit crop health and productivity [53].

The exploration of genetic resistance to RKN across cucurbit crops highlights a critical gap in current agricultural practices. Of the cucurbits, some zucchinis (*C. pepo*) show resistance to *M. incognita,* exhibiting a Zeck index score of 4.3 [55]. Nonetheless, the genetic basis of this resistance remains unknown and may be of a quantitative nature rather than governed by a major dominant gene [55,56]. In an evaluation using recombinant inbred lines (RIL) from specific crosses, we identified zucchini lines that significantly inhibit *M. incognita* reproduction by over 90%. However, these RILs remained susceptible hosts for *M. javanica* [57]. Currently, a recessive gene (*mj*) from *C. sativus* var. hardwickii provides limited resistance, but it is not present in commercial cultivars [58,59]. In various plant species other than cucurbits, genes that confer resistance to nematodes, particularly those triggering the hypersensitive response (HR) in root cells, have been identified. The *Mi* gene in tomatoes is a well-characterized example, providing resistance to three species of RKN [60]. Similar early HR responses are seen in coffee with *Mex-1*-mediated resistance [61], in black pepper with *Me3* [62], and in soybeans during incompatible interactions [63]. While genetic resistance to root-knot nematodes in cucurbit crops, including pumpkins, has not been widely identified [50], evaluations of root-stock candidates for grafting, such as kumati kai, African horned cucumber, and pumpkin, have exhibited enhanced resistance to RKN [64]. This insight highlights the potential of integrating genetic resistance into cucurbit breeding programs. The identification and utilization of RKN-resistant rootstocks for grafting could pave the way for innovative strategies to combat RKN infestations, thereby enhancing the resilience and productivity of cucurbit crops.

The oxidative stress response of *C. moschata* upon nematode infection presents a sophisticated biological phenomenon that underscores the complexity of plant–pathogen interactions. Our analysis demonstrates a significant increase in H_2_O_2_ levels, indicative of the activation of the plant’s defense responses, while levels of O_2_^•−^ and the marker of lipid peroxidation, MDA, did not exhibit significant deviations from the control (Figure 3). In the susceptible tomato cultivar *Solanum lycopersicum* L. cv. Zheza 205, it was shown that *M. incognita* infection led to an increase in MDA levels [65], while detection in the leaves from separate research showed that MDA levels decreased in the leaves infected with *M. javanica* [66]. This suggests a targeted activation of ROS signaling pathways, potentially reflecting a sophisticated antioxidative response rather than a generalized oxidative stress reaction [67]. ROS, though causing extensive cellular damage, also play a pivotal role in defense signaling and the establishment of microbial antagonism. The observed increase in H_2_O_2_ levels aligns with the plant’s defense arsenal, which is crucial for countering pathogenic invasions [68]. Additionally, studies on tomatoes infected with *M. incognita* have shown that furostanol glycosides from *Dioscorea deltoidea* can mitigate oxidative stress by modulating lipid peroxidation, suggesting a potential avenue for enhancing *C. moschata*’s resilience to nematode-induced stress through similar biochemical mechanisms [69]. The specific modulation of ROS observed, characterized by an increase in H_2_O_2_ without a corresponding rise in O_2_^•−^ and MDA levels (Figure 3), implies a selective antioxidative strategy possibly orchestrated by the plant to mitigate the oxidative stress while still mobilizing defense mechanisms against the nematode threat. This selective ROS response aligns with the broader understanding of ROS as dual-function molecules within biological systems, acting both as signaling molecules that activate defense responses against pathogens, including programmed cell death, and as hypersensitive responses to contain and neutralize pathogen spread at the infection site [70,71].

In the intricate interaction between plants and PPNs, our analysis has illuminated a crucial component of plant defense mechanisms, as evidenced by the observed increase in GSH levels (Figure 4). This elevation in GSH may be indicative of an adaptive response by the plant to counteract heightened oxidative stress. Glutathione, a pivotal antioxidant, plays an essential role in maintaining cellular redox homeostasis, thereby safeguarding the plant cell from oxidative damage induced by nematode infection [72,73]. Interestingly, the ASC levels did not exhibit a significant change, suggesting a nuanced selective upregulation of components within the plant’s antioxidant defense system in response to nematode attack. This differential regulation suggests a sophisticated defense strategy where plants may prioritize the activation of specific antioxidants based on the nature of the stress encountered. The complex scenario underscores the ASC–GSH pathway’s significance in redox regulation during plant–PPN interactions.

Plant antioxidative enzymes play a crucial role in the interaction between root-knot nematodes and plants. Extensive research into plant–nematode interactions has highlighted the pivotal role of antioxidative enzymes [74]. These systems are integral to the physiological responses of plants during pathogenic incursion and similarly critical for nematode endurance in conditions characterized by elevated ROS levels, which can lead to oxidative stress [75]. Enzymes such as SOD, POD, and CAT, etc. remove free radicals and activated oxygen species, which are essential for plant defense mechanisms during pathogen attacks [76]. Studies have shown that resistant genotypes of plants infected with root-knot nematodes exhibit higher SOD activity compared to susceptible genotypes, indicating a protective response against nematode infection, whereas CAT activity in resistant genotypes decreases upon infection [77,78,79]. Conversely, CAT activity was shown to be elevated upon *M. javanica* infection [66], suggesting variances in antioxidant enzyme responses between resistant and susceptible plants. In a study comparing sweet potato cultivars resistant to and susceptible to RKNs, it was found that although both resistant and susceptible cultivars demonstrated an increase in SOD activity, resulting in elevated H_2_O_2_ levels, the susceptible cultivars exhibited higher CAT activity, leading to reduced H_2_O_2_ levels in the initial stages of infection. However, as the infection progressed, H_2_O_2_ levels in these susceptible cultivars increased [75]. Interestingly, in our study, while specific activities of GR and APX are significantly reduced in groups infected with *M. incognita* compared to mock controls (Figure 5), the activities of SOD, GR, CAT, and POD, when quantified per fresh weight (Figure S1), are significantly induced. A notable 2.0-fold increase was observed in protein levels of *M. incognita*-infected pumpkins compared to the mock control group (Figure 5A). Whereas tomato leaves showed a lower protein concentration upon *M. javanica* infection [66], similar observations were made in studies on bitter gourd, where a substantially higher rate of protein synthesis during infection was noted [80]. This global protein enhancement may reflect the activation of other defense pathways or a generalized increase in protein synthesis as part of the plant’s stress response [67]. Protein profile assessments via SDS-PAGE analysis revealed the presence of variances in SOD, APX, and POD (Figure 7). However, quantification of the different isoforms would require further analysis utilizing methods such as fast atom bombardment, electrospray ionization, or matrix-assisted laser desorption and ionization.

## 5. Conclusions

Our study illuminates the physiological responses of *C. moschata* to *M. incognita* infection, characterized by stunted growth and an orchestrated increase in antioxidant defense. These insights not only provide a foundation for understanding the biochemical landscape of nematode–plant interactions but also highlight potential targets for enhancing nematode resistance in Cucurbitaceae crops. Research on modulating ROS activities and antioxidative enzymes in plant–nematode interactions provides a basis for developing innovative approaches to enhance plant defenses against RKNs, leading to sustainable management of nematode diseases in agriculture. The variability influenced by the type of nematode, plant species, and cultivar’s resistance level underscores the complexity of this interaction, while unraveling these dynamics offers valuable insights into effective strategies for managing nematode infestations, potentially leading to more resilient crop varieties and improved yield outcomes.

## Data Availability

The original contributions presented in the study are included in the article; further inquiries can be directed to the corresponding author.

## Supplementary Materials

The following supporting information can be downloaded at: https://www.mdpi.com/article/10.3390/biology13040267/s1. Figure S1: The effect of *Meloidogyne incognita* on antioxidative enzyme activities in *Cucurbita moschata*.
