# Supplementary material for: Antioxidant Responses and Growth Impairment in Cucurbita moschata Infected by Meloidogyne incognita
Source: Biology (Basel). 2024 Apr 16;13(4):267. doi: 10.3390/biology13040267 (PMC11048190; doi:10.3390/biology13040267)
Supplement: Supplementary file 1 [file biology-13-00267-s001.zip › biology-2952533-supplementary.pdf]

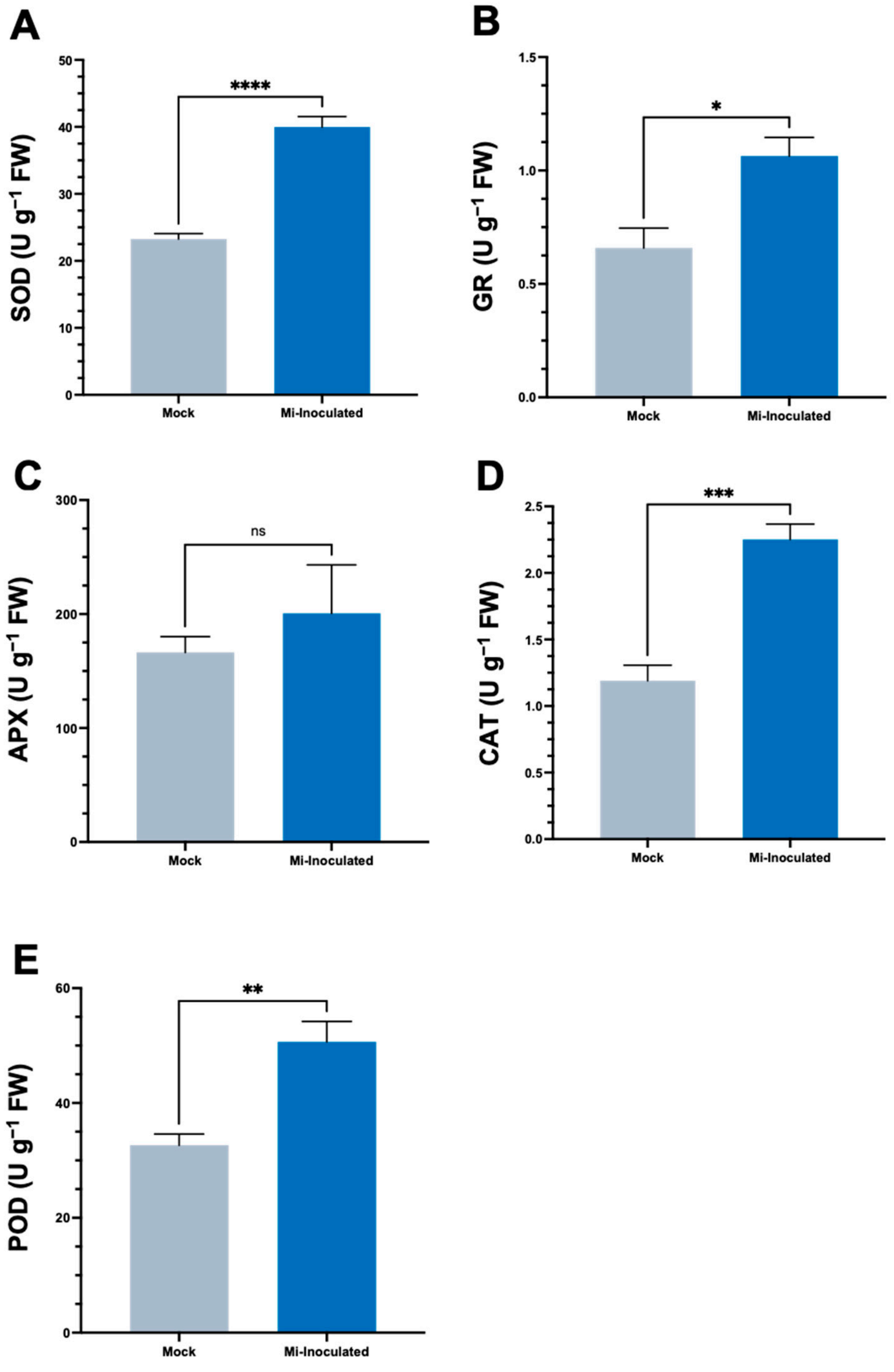

Figure S1. The effect of *Meloidogyne incognita* on antioxidative enzyme activities in *Cucurbita moschata*. Superoxide dismutase (SOD) (**A**), glutathione reductase (GR) (**B**), ascorbate peroxidase (APX) (**C**), catalase (CAT) (**D**), and peroxidase (POD) (**E**). *Meloidogyne incognita*-inoculated plants are labeled as Mi-inoculated. The values represent mean ± SE (n = 5); * indicates $p \leq 0.05$; ** indicates $p \leq 0.01$; *** indicates $p \leq 0.001$; ****indicates $p \leq 0.0001$; and ns denotes no statistical significance. Student's *t* test comparing groups as indicated.
